# Sex Differences in Physiological Acclimatization after Transfer in Wistar Rats

**DOI:** 10.3390/ani4040693

**Published:** 2014-10-30

**Authors:** Johanna W. M. Arts, Klaas Kramer, Saskia S. Arndt, Frauke Ohl

**Affiliations:** 1 Department of Animals in Science & Society, Division of Animal Welfare & Laboratory Animal Science, Veterinary Faculty, Utrecht University, Utrecht, 3584 CM, The Netherlands; E-Mails: k.kramer@vu.nl (K.K.); s.s.arndt@uu.nl (S.S.A.); f.ohl@uu.nl (F.O.); 2 Harlan Laboratories B.V., P.O. Box 553, Venray, 5800 AN, The Netherlands; 3 Department of Occupational Health, Safety and Environment, Free University, Amsterdam, 1081 HV, The Netherlands

**Keywords:** transportation, transfer, acclimatization, heart rate, blood pressure, locomotor activity, corticosterone, rat

## Abstract

**Simple Summary:**

This study in laboratory rodents shows a sex specific effect of breeder to research facility transfer on several physiological parameters, such as heart rate and blood pressure. We recommend at least 8 days of acclimatization time after transfer in male rats and at least two weeks in female rats, before using these animals in research.

**Abstract:**

Most laboratory animals used in research are vendor-bred and transferred to research facilities. Transfer procedures might have considerable and unintended effects on research results. In the present study we compared physiological and behavioral parameters before and after external and internal transfer, as well as between transferred and non-transferred Wistar rats. The impact of both external and internal transfer on body weight, plasma corticosterone levels, heart rate, blood pressure, and locomotor activity was studied in both male and female Wistar rats, taking into account the sex differences in stress responsivity. External transfer was found to decrease body weight, increase plasma corticosterone, increase activity, increase heart rate in female rats, but decrease heart rate in male rats. Parameters showed differences between the sexes and light phases. This study shows that acclimatization after transfer is sex-specific and researchers should take the sex into consideration when determining the acclimatization period. It is recommended to allow for acclimatization of at least 8 days in males and two weeks in females after external transfer and timely (2 days before starting experiments) transfer the animals internally to the testing room.

## 1. Introduction

Most laboratory animals used in research are vendor-bred and transferred from the breeding facility to a research facility at some point. During such a transfer procedure, animals are exposed to numerous unfamiliar environmental influences, such as noise and smells, changes in temperature and light conditions, and frequent handling and movement [1]. Transfer to the research facility may last several days, thus representing a prolonged series of diverse stressors for the animals, which can be expected to induce a significant stress response in the animal [2,3]. Also in-house transfer has been found to have long-lasting effects [4]. The use of stressed animals in experiments is considered to have considerable and unintended effects on research results, and results obtained in stressed animals are known to differ significantly from those in non-stressed animals [5,6,7]. Even if animals are not vendor-bred, they are usually transferred from a breeding section to a research section in-house, which may affect the animals just as well [3].

In general, animals subjected to a stressful procedure like transfer between different locations react with diverse physiological responses, such as alterations in body weight, hormone and glucose levels, heart rate, and blood pressure [8,9,10,11,12,13,14]. Although no previous studies have embraced all parts of the transfer procedure, some studies have found deteriorating effects of transportation or transfer on the immune system [15,16] and on nutritional parameters [12]. However, reliable and reproducible scientific results from experiments using laboratory animals are demanded to represent baseline values or experimentally induced alternations from such baseline values [5,6,7] and, thus, physiological status after transfer at the beginning of each experiment must be (re)stabilized up to a level that does represent such a baseline. It should be noted though that baseline levels after transfer are not necessarily identical to levels before transfer (such a fixed baseline status is referred to as homeostasis [6,7], but instead may be established at a different, yet stable, level in response to previous experiences and age-related changes of the individual (such a dynamic baseline status is referred to as allostasis) [6,7].

In biomedical research, recommendations for acclimatization periods vary grossly: the majority of the researchers working with laboratory animals wait 5–7 days after transport from the breeders to their animal facility before using their laboratory animals in experiments, while a diverse set of recommendations can be found, varying from some days to 2–3 weeks [8,10,17,18]. Given that allostatic processes in response to prolonged stress procedures have been shown to last from hours up to weeks and longer [19], transfer-induced changes may represent an important confounding variable during a time period that potentially exerts commonly used acclimatization periods. Therefore, the question how long animals should be allowed to acclimatize after transfer deserves further investigation [9]. Surprisingly few studies have fully explored the acclimatization period that might be necessary after transfer of research animals [9,12,13]. These studies, however, primarily have been concentrating on the effects of transport alone, while not experimentally controlling for all parts of the transfer procedure. We therefore set up a series of experiments aiming at determining the duration of stress responses in male and female rats during and after having been transferred between two locations. It is important to note that we tried to stay as close to the daily practice as possible, even if that restricted our parameters. The transfer of research animals withholds a multitude of procedures, which cannot be investigated independently. Therefore, the transfer-procedure as described in our studies consists of weighing and packing the animals, multiple movements of the transportation boxes between buildings/areas (breeding location—barrier-holding area, where animals arrive after leaving the barrier—logistic center, where transportation boxes are distributed onto the trucks—research facility—in-house) and includes the change of environment and caretakers. In a previous study we measured a variety of physiological and behavioral parameters in male Wistar rats before and for 3.5 weeks after transfer from a supplier to a research facility [8]. We found that heart rate, mean arterial blood pressure, locomotor activity, plasma corticosterone (CORT), and certain behavioral parameters did not return to pre-transport levels during this time period. Thus, stabilization seemed to occur on an allostatic level rather than a homeostatic level. In our previous transportation study, heart rate, blood pressure, and behavioral parameters were stabilized after approximately one week, corticosterone had not been stabilized after 3 weeks [8].

To determine external validity of the previous study, we performed an extended replication in the current study, changing the starting location, adding the factor of sex and adding an additional internal (in-house) transfer to the study as well as prolonging the measuring period after transfer [20,21,22,23,24].

To reliably acquire blood pressure, heart rate, and activity, radio-telemetry transmitters were used. Radio-telemetry is a method to obtain accurate and reliable physiological measurements from conscious, freely moving animals [21,22] over a longer period of time [14,25,26,27,28]. In addition, body weight and blood corticosterone (indicating acute hormonal stress response) were measured [6,7].

## 2. Animals, Materials, and Methods

### 2.1. Ethical Statement

The protocol of the experiment (DEC-DGK number: 2008.I.01.004) was peer-reviewed by the scientific committee of the Department of Animals in Science & Society, Utrecht University, The Netherlands, and approved by the Animal Ethics Committee of the Utrecht University, The Netherlands.

### 2.2. Animals and Housing

Subjects were 18 male and 18 female rats (HsdCpb: WU; Wistar Unilever, Harlan Laboratories BV, Horst, The Netherlands), 3 weeks old (50–60 g) at the start of the experiment. Animals were weaned and moved to the animal room of the Surgical Unit, adjacent to the animal breeding unit. They were randomly divided over 18 Makrolon Type III cages, with a floor surface of 820 cm^2^ (Tecniplast, S.p.A., Buguggiate, Italy) and Aspen bedding (Abedd, Indulab Switzerland), enriched with homemade square PVC piping shelters and paper tissues (Kleenex, Kimberley-Clark Inc., Madrid, Spain), containing either two males or two females and placed in an individually ventilated cage (IVC) rack (type AHM A4, Biozone Ltd., Ramsgate, UK).

Because of the restrictions of doing telemetry implantation surgery and performing an experiment inside the breeder’s animal barrier, the animals used in this experiment grew up in the surgical unit adjacent to the barrier instead of inside the barrier as in the former study, regarding all normal breeding unit protocols. Nevertheless, circumstances were somewhat different in the surgical unit, especially concerning the amount of animal caretakers (less) and the working activities during the day (less) and therefore the total amount of disturbance during the entire housing period (less).

During the first part of the experiment, until transfer, all animals were housed in the surgical unit of the breeder in an animal room with a controlled environment maintained at a constant temperature of 20 °C (19.5–22 °C) and a relative humidity of >40% (42%–55%). Animals had free access to food (Harlan Teklad irradiated 18% protein rodent diet 2918) and processed water (acidified (pH 5.8–6.4), chlorinated (6–8 ppm), softened, and filtered (0.02 microns)). Light-dark regime was maintained at 12:12h, lights on at 6:00 am, ±350 lux one meter above the floor. The animals remained in the same group composition during the entire experiment. Cage cleaning and weighing of the animals occurred weekly. All procedures were performed by either the researcher herself or by the biotechnical staff at the facilities.

According to a randomized schedule, five cages with males were selected and assigned to Group 1, the remaining four cages were assigned to Group 3. Five cages with females were randomly selected and assigned to Group 2, the remaining four cages were assigned to Group 4.

### 2.3. Blood Sampling

**Figure 1:**
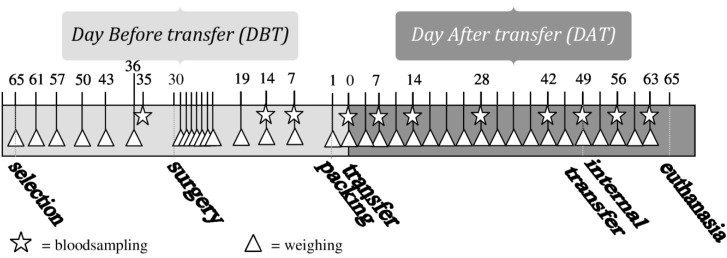
Timeline of performed procedures.

Blood samples were taken at 11 predefined moments during the experiment, always on weekdays at 3–4 h after “lights on” (see Figure 1). The first blood (BS1) sample was taken 5 days before surgery, the second one (BS2) 16 days after surgery. Blood Samples 2–6 (BS2-BS6) were taken weekly in the period of 14 days before until 14 days after transfer, with Blood Sample 5 (BS5) taken on the day of transfer, directly after unpacking in the animal facility. Blood Samples 7 (BS7) and 8 (BS8) were taken after periods of 2 weeks on Days 28 (DAT28) and 42 (DAT42) after transfer. BS9 was taken directly after internal transfer to a different animal room (see “internal transfer”), BS10 and BS11, one and two weeks after internal transfer, respectively. The first blood sample was taken from all animals. The other blood samples were taken from one animal per cage, according to a randomized schedule with equal variation between animals with and without transmitters.

Blood was sampled from the lateral tail vein, using a sterile razorblade and microvette tube, as described by M. Fluttert *et al*. [29]. The samples were taken in a separate, dedicated room, within 2 min of retrieving the cage from the rack in the animal housing room. Samples were immediately put on ice and within an hour brought to the lab to be centrifuged (10 min, maximum RPM). Plasma was pipetted into 1.5 mL Eppendorf tubes and stored at −18 °C until analysis.

Plasma was analyzed with a commercial radioimmunoassay (RIA) kit on plasma CORT, specified for rats and mice (ImmuChem^TM^, MP biomedicals, LLC, Costa Mesa, CA, USA) according to the protocol of the supplier. Each sample was analyzed in duplicate.

### 2.4. Telemetry

Surgery took place at the Surgical Unit of the breeder (Harlan NL, Horst, the Netherlands), thus ensuring that no animals were transported prior to the study. When the animals were 8 weeks old (average body weight males 300 g, females 197 g), surgery was performed by an experienced surgical technician on eighteen animals. In all 18 cages, one animal was implanted with a 7.7 g radio-telemetry transmitter (model TA11PA-C40, Data Sciences International (DSI), St Paul, MN, USA). Pre-operatively the animals were given analgesia with carprofen (Rimadyl^©^, Pfizer Animal Health, Exton, PA, USA) subcutaneously, 5 mg/kg), which was repeated every 12 h until 2 days after surgery. The animals were anesthetized with isoflurane inhalation anesthesia. During surgery, the eyes were protected with Duodrops^®^ (Produlab Pharma, Raamsdonkveer, The Netherlands). The implantation procedure was carried out under strictly aseptic conditions as described by Kramer *et al*. [24] for mice and by Huetteman [30] for laboratory rodents, in which the transmitter cannula was directly fixed into the abdominal aorta, and the body of the transmitter was permanently fixed to the inside of the abdominal muscles. The still unconscious animals were brought back to the original animal room, where they were placed in a cage that was placed on a heating pad (approximately 37 °C). As soon as the animals regained consciousness after the surgical procedure and were able to walk, they were pair housed with their non-implanted cage mate. All cages were placed above a receiver station. To prevent detection from adjacent transmitters, all receivers and cages were placed in a U-shaped stainless steel profile. During the first week after surgery the animals were weighed and checked daily. Directly after surgery, telemetry measurements started at a collection rate of every 3 m for 10 s, 24 h per day. Locomotor activity was expressed as counts per minute, with every count representing a movement of the rat of 1.5–2 cm, detected by the system as change in signal strength. Locomotor activity was recorded continuously and was stored every 3 min (total activity of 3 min).

### 2.5. External Transfer Procedure

Transfer in this experiment includes not only the physical movement of the animals from breeder to research facility, but also the packing procedure and changes of animal room, cages, and caretakers.

Thirty days after surgery, the rats of Group 1 and 2 were prepared for transfer into the room in which they were housed. The rats were removed from their cage, weighed, and allocated with their cage mate in solid floor plastic transport boxes with filters (64 × 42 × 16 cm, Williton Box Co., Taunton, UK). The boxes were prepared with wood shavings (Tierwohl, Classic^®^ bedding, Rettenmaier & Söhne, Rosenberg, Germany), weighed diet pellets on the box floor (Harlan Teklad Rodent Diet 2018), and weighed Hydrogel™ sacks (Harlan, Indianapolis, IN, USA) as a water source. The lids of the boxes were closed and taped. The animals of Group 3 and 4 were left undisturbed.

The animals in Group 1 and 2 were packed in the transport boxes between 10.00 a.m. and 11.00 a.m. After 1 h, the boxes were brought to a second unit (holding area) where all boxes with animals were collected. At the end of the afternoon (around 16.00), all boxes were transported by truck to a logistic center (approximately 30 km from breeding site). The animals were kept there overnight, until the loading of the vans occurred. The temperature in the holding area was set to 17 °C. The next day at 04.00 a.m. the animals of Group 1 and 2 were loaded into an unlit, climate-controlled (15 °C) van and transported to the receiving facility (Central Laboratory Animal Research Facility Utrecht (CLARF), Utrecht, the Netherlands). The journey lasted approximately 6 h, including multiple stops for delivery of other animals. As soon as the transportation boxes with animals arrived at the receiving facility, they were placed on the telemetry receivers in the designated animal room for 2 h before they were unpacked by the researcher. At the receiving facility, data recording resumed every 3 min for 10 s, 24 h per day for 65 days. Environmental and housing conditions were comparable to those in the breeding facility. Consumed amounts of feed and Hydrogel were acquired by deducting amounts left in the box from the initial amounts.

### 2.6. Internal Transfer Procedure

At the 49th day after transfer (DAT49), the transported groups (Group 1: male and Group 2: female) were internally transferred to another animal room. The cages with the animals were placed on a trolley and left on this cart for approximately half an hour, while the telemetry setup was installed in the new animal room. Then they were driven around for approximately 5 min. In the new animal room, the cages were positioned in a new randomized order in the racks and telemetry measurements continued for two more weeks. Figure 1 shows an overview of all performed procedures.

### 2.7. Statistical Analyses

In the present study the cage was the experimental unit [31]. Therefore, body weight (gain) was averaged per cage. For analysis of plasma CORT of BS1, cage-averages were also used. This did not apply to the remaining blood samples, because then only one animal was sampled. Data of the telemetry output was averaged to 24 h or light-period per animal. Some plasma samples had CORT values below the detection limit. These data values were replaced by the value pre-half of the detection limit [32]. CORT was analyzed using the area under the curve (auc) [33].

Data was imported into Excel (Microsoft Inc. Seattle, USA) for sorting and then exported to SPSS^®^ for Windows (version 20) software program (SPSS Inc., Chicago, IL, USA). Two-sided, exact (*i.e.*, for the non-parametric tests [34,35,36,37]; probabilities were estimated throughout. Continuous data (body weight, blood plasma parameters, and telemetry parameters) were summarized as means and standard deviations (SD). Telemetry data was analyzed both by whole day (24 h) and split for light period (average 12 h dark period and 12 h light period). One of the big advantages of telemetry is that not only groups or experimental units can be compared, but also the animals can be their own control. This was used to make before transfer *vs.* after transfer comparisons within the groups. Surgery was found not to have an effect on the results and was therefore left out of further analyses.

Data that did not show Gaussianity was transformed if possible, if not, the continuous parameter in question was rank-transformed [38]*.*

Continuous data were tested for significant differences by multivariate repeated measures ANOVA. Tests of significance were derived using the Wilk’s lambda criterion [39]. For telemetry data within the groups (to compare the animal with itself), a period of seven days before transfer was compared with an equal period after transfer, again using a repeated measures ANOVA (within group effects: time, lightphase). It is known that noise and activities in animal facilities have influence on physiological values of laboratory animals [40]. Therefore, before-after analyses were performed by comparing only equal days (for example Monday before *vs.* Monday after), ensuring that no effect of day of the week could have biased the results. Between-group differences (Sex, Treatment, Sex and Treatment-interaction) were analyzed by comparing the groups with a repeated measures ANOVA during the different phases of the experiment (*i.e.*, before transfer, which is the baseline or reference period, between day before transfer (DBT)15 and DBT2; and after transfer between day after transfer (DAT)1 and DAT48), as well as during before (DAT41-DAT48) and after (DAT50-DAT62) internal transfer.

For all repeated measures ANOVA’s, homoscedasticity was tested by the Levene’s test [41]. When necessary, the variances were equalized by logarithmic or logistic transformation of the continuous data or by rank-transformation [38].

If the repeated measures ANOVA detected significant effects, group means of the continuous parameters were further compared. Between-subject *post hoc* comparisons (group differences) were done with either unpaired Student’s t tests for normally distributed data, or for non-normally distributed data, the same comparisons were performed using a Mann-Whitney U-Wilcoxon rank sum W test. The unpaired Student’s t tests were performed using pooled (for equal variances) or separate (for unequal variances) variance estimates. Within-subject *post hoc* comparisons (time effects, *i.e.*, before and after packing/transfer) were made using a paired Student’s t test when the difference between the two compared groups was normally distributed or, if not, Wilcoxon matched-pairs signed ranks test was used [42].

To take the greater probability of a Type I error due to multiple hypotheses into account, a more stringent criterion should be used for statistical significance. We approached this problem by calculating Dunn-Šidák corrections (α = 1 − [1 − 0.05]^1/λ^; λ = number of comparisons) [43]. In all other cases, the probability of a Type I error <0.05 was taken as the criterion of significance.

## 3. Results

### 3.1. Body Weight and Feed Consumption

Both body weight and body weight gain (ranked) showed an overall significant time × sex effect (repeated measures: all *p* < 0.001).

On the day of transfer (DAT0), there was a significant difference in body weight gain between the transferred and the non-transferred groups in both male and female animals (males: t_(16)_ = −2.34, *p* = 0.032; females: t_(16)_ = −5.64, *p* < 0.001). Male transferred rats decreased 1.12 percent in body weight between packing and unpacking, male control rats increased 0.75 percent in body weight during the same period. Female transferred rats decreased 2.37 percent in body weight between packing and unpacking, female control rats increased 0.43 percent in body weight during the same period.

During transfer male rats consumed on average 51 (41–68) gr Hydrogel and 17.3 (14–23) gr feed, female rats consumed on average 54 (38–74) gr Hydrogel and 7.5 (5–12) gr feed.

Internal transfer did not have a significant effect on body weight or body weight gain, but sex differences remained.

### 3.2. Plasma Corticosterone (CORT)

**Figure 2:**
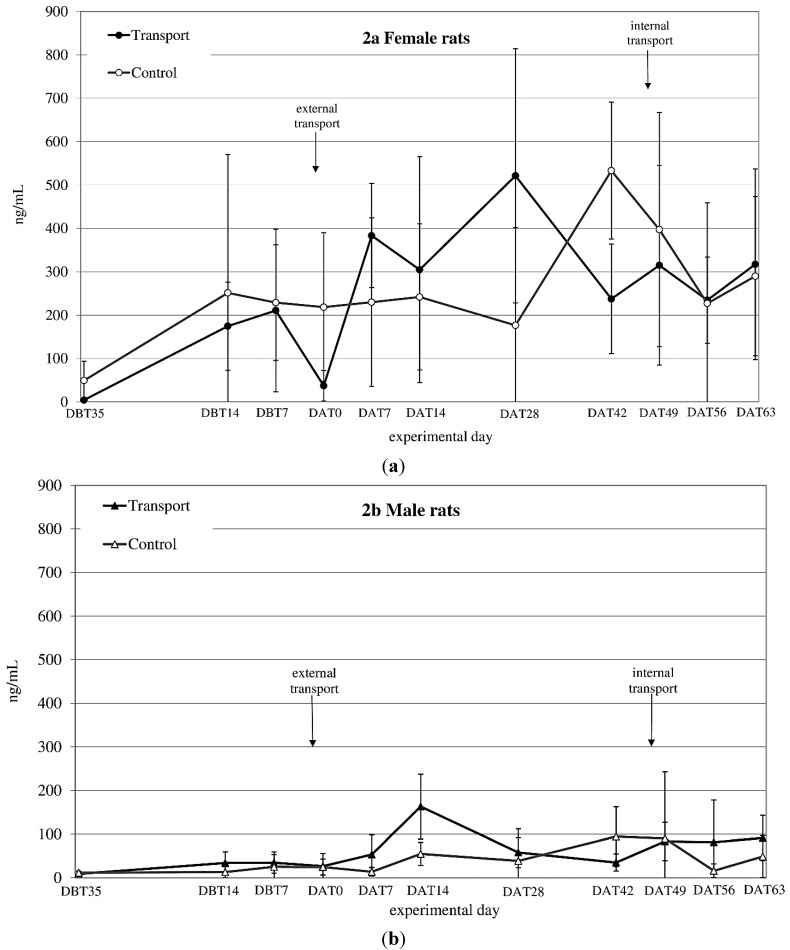
Averaged plasma corticosterone (CORT) levels in ng/mL (mean ± SD) at 11 sampling moments in female (**a**) and male (**b**) Wistar Unilever (WU) rats, DBT: day before transfer, DAT: day after transfer (TP: n = 5, CO: n = 4).

CORT_auc_ both before and after transfer showed a significant time × sex effect (before transfer: F_before(1,14)_ = 6.1; *p* = 0.027 after transfer: n.s.) (see Figure 2a,b). CORT_auc_ before *vs.* after transfer also showed a sex effect (females > males; F_(1,8)_ = 43.33; *p* < 0.001).

CORT_auc_ showed a sex effect before and after internal transfer: (both Females > Males; F_before(1,8)_ = 24.45; *p* = 0.001, F_after(1,8)_ = 55.56; *p* < 0.001). Comparing CORT_auc_ measurements before with measurements after internal transfer showed a before-after (before transfer > after transfer; F_(1,8)_ = 13.00; *p* = 0.007) and a sex-effect (females > males; F_(1,8)_ = 74.15; *p* < 0.001).

### 3.3. Heart Rate

**Figure 3:**
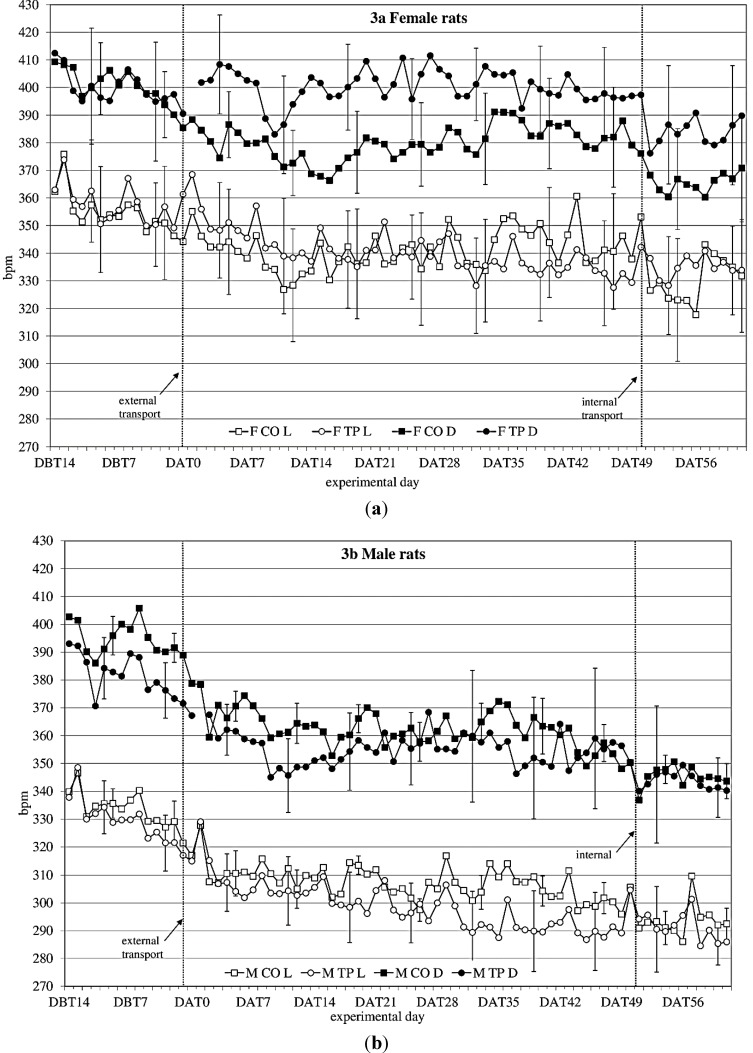
Average 12 h heart rate in beats per minute (bpm) (mean ± weekly standard deviation (SD)) during dark(D) and light(L) period between DBT14 and DAT61 in transported (TP) and control (CO) female (**a**) and male (**b**) WU rats. DBT: day before transfer, DAT: day after transfer (TP: n = 5, CO: n = 4).

During the baseline period (DBT15-DBT2), heart rate (Figure 3a,b) showed a time × sex × transfer-interaction trend (F_(11,352)_ = 1.60; *p* = 0.098) on heart rate. Splitting for light phase showed a time × sex × transfer effect (F_(10,140)_ = 1.93; *p* = 0.045), showing that over time, transfer affects heart rate in the two sexes differently.

After transfer (DAT2-DBT48), heart rate showed a sex effect (females > males; F_(1,32)_ = 10.76; *p* = 0.003). Splitting for light phase showed during the dark period a time × transfer (F_(8,107)_ = 2.2; *p* = 0.036) effect and a sex × transfer (F_(1,14)_ = 4.1; *p* = 0.063) and a time × sex × transfer (F_(8,107)_ = 1,76; *p* = 0.096) trend, suggesting that during the dark period transfer affects heart rate differently in the two sexes, depending on the time. During the light period it showed a time × sex (F_(28,393)_ = 2,26; *p* < 0.001) and a time × transfer (F_(9,393)_ = 4.82; *p* < 0.001) effect.

Comparing the week before with the week after transfer showed a significant before-after × sex effect (dark period; F_(1,14)_ = 14.37; *p* = 0.002 light period; F_(1,14)_ = 11.79; *p* = 0.004) during both dark- and light period.

Comparing the groups (transferred *vs.* control) per week showed sex effects in heart rate every week. There were sex × transfer effects in the first 4 weeks after transfer during dark period.

Comparing the week before internal transfer to the week after internal transfer, the transferred group showed a significant sex-effect (females > males; dark period: F_(1,8)_ = 10.63; *p* = 0.012; light period: F_(1,8)_ = 20.20; *p* = 0.002) and a time effect (dark period: F_(4,33)_ = 2.71; *p* = 0.045 light period: F_(5,43)_ = 4.31; *p* = 0.002) during both dark and light periods. During the dark period there was also a before-after transfer effect (before transfer < after transfer; F_(1,8)_ = 7.44; *p* = 0.026).

### 3.4. Mean Arterial Blood Pressure

Unfortunately, due to unexpected technical errors with data sampling no correct mean arterial pressure data was recorded at the starting/control facility. Therefore we have only reliable mean arterial pressure data of the transferred animals after transfer. Telemetry data after transfer showed no significant sex differences in mean arterial pressure and no significant effects of internal transfer on mean arterial pressure.

### 3.5. Activity

**Figure 4:**
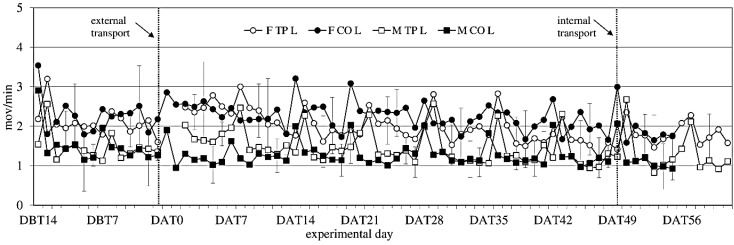
Locomotor Activity (ACT) (mean ± SD) during the light period in days −14 (DBT14) to 62 (DAT62) in transported and control male and female WU rats. DBT: day before transfer, DAT: day after transfer (TP: n = 5, CO: n = 4).

Activity showed no significant difference between the experimental groups before transfer (Figure 4). After transfer activity showed a sex-effect on activity during the light period (females > males; F_(1,14)_ = 14.34; *p* = 0.002), but not during the dark period.

Comparing activity the week before to the week after external transfer showed a sex-effect (females > males; F_(1,14)_ = 12.13; *p* = 0.003) during the light period, and a before-after × transfer interaction (in the transferred groups: before < after; F_(1,14)_ = 10.55; *p* = 0.006) during the dark period, meaning transfer increased activity. Comparing the groups (transferred *vs.* control) per week showed overall sex effects during the light period.

Comparing the week before internal transfer to the week after, internal transfer showed a sex-trend (females > males; F_(1,8)_ = 4.04; *p* = 0.079) in the transferred group during the light period. It also showed a time effect during the light period (F_(5,37)_ = 14,60; *p* < 0.001) and a time trend during the dark period (F_(4,32)_ = 2.48; *p* < 0.064). There were opposite before-after effects of internal transfer on activity during the dark and light periods. During the dark period there was a before-after effect, with before transfer > after transfer (F_(1,8)_ = 5.28; *p* = 0.051), and during the light period the effect was before transfer > after transfer (F_(1,8)_ = 5.87; *p* = 0.042).

### 3.6. Temperature and Humidity in Transportation Boxes

Temperature logging inside the transportation boxes showed a highly variable temperature during transfer. Temperature fluctuated between 18.8 °C and 25.5 °C between packing and unpacking in spite of the climate control of the facilities and the van. Average humidity in the transportation boxes rose to levels of 84%, in contrast to normal housing conditions of 40%–50%.

## 4. Discussion

Transfer of animals between facilities includes a variety of factors. This multitude of factors makes it difficult to standardize the transfer process and, thus, respective effects on transferred batches of animals may vary considerably. Therefore, it is not surprising that the results of the present study did not exactly reproduce our previous findings on the effects of transfer in male rats [8]. Still, we again found long-lasting changes at the physiological and activity level in male Wistar rats and, moreover, the sex comparison revealed some marked differences in transfer effects between male and female rats.

While both males and females lost weight during transfer, this loss of body weight was larger in females, similar to the effects found by Sterrenburg *et al*. [44]. Weight loss, however, was modest compared to other transfer studies [13,14,45], a difference that may be due to the fact that total transfer procedures are unlikely to be identical in different studies. The relatively modest weight loss in the present study may be explained by decreased food and water intake and increased defecation during transfer [4,46,47], since BW gain returned to control levels within two days. Body weight sometimes is used as guiding indicator for determining the necessary acclimatization period of animals after transfer [45]. Given, however, the marked differences in body weight changes in response to apparently different overall transfer procedures and, moreover, that body weight seems to stabilize earlier than other parameters, it might not be advisable to rely on this parameter alone. Blood corticosterone (CORT) levels revealed a large variation in both sexes, but especially in females, making it difficult to interpret this data straight forwardly. Both sexes showed an increase in CORT 1 week after transfer (Figure 2a,b). After subsequent internal transfer no significant increase in CORT was observed. This finding is in contrast with the results of a study by Tuli *et al*. [4], who found a one-day rise in CORT in BALB/c mice after in-house transfer. However, BALB/c mice have been reported to show a high, acute stress response to environmental challenges [48], while Wistar rats have not been reported to be especially susceptible to stressors [49]. Finally, in non-transported males as well as females an increase in CORT levels was found in experimental Week 4 (DAT42) after transfer, which could not be explained by known external factors.

Large variability and higher CORT levels in females compared to males like those obtained in this study have been reported before by Van Ruiven *et al*. [12], where female CORT levels were increased compared to those in males several days after transfer. Similarly, Sterrenburg *et al*. [44] and García-Cáceres *et al*. [50] reported female Wistar rats as having higher CORT levels than male rats. Estrus is one known factor that causes high variation in basal CORT, resulting in female animals having high(er) CORT levels than males and decreased negative feedback ability [21,51,52,53,54,55,56,57]. The decreased CORT levels in transferred females directly after transfer is in line with findings of Van Ruiven *et al.*, who similarly found lowered CORT-levels after transport and increased levels 3 days thereafter [12]. Such changes may either be due to effects on the feedback mechanism of the hypothalamic-pituitary-adrenal (HPA)-system, as mentioned above, but may as well be caused by fasting or heat stress of the animals during transfer [58,59]. The high variability in CORT levels in female rats makes CORT a rather unreliable parameter for assessing the effect of transfer. Further, we decided not to correct for cycle to decrease the inter-individual CORT variability, because this would have demanded additional handling of the animals, which would make the study less representative for daily practice.

In conclusion, a stable baseline in CORT values did not seem to be established in either male or female rats throughout the experimental period.

Overall, females were more active than males, a sex difference that is well known in rats [60,61]. Both sexes showed weekly activity peaks associated with cage cleaning. External transfer increased activity levels in both males and females for 8 days, before returning to before-transfer levels. Internal transfer briefly decreased activity levels in both sexes, which returned to previous baseline levels at Day 2 after internal transfer, indicating acclimatization periods of 8 days after external and 2 days after internal transfer.

The highly variable temperature (rises of six degrees within one hour) and high humidity inside the transportation boxes during the entire transfer procedure can be considered one of the main stressors for transferred animals [62], causing a rapid ammonia production [63], although in the present study transportation-boxes contained only two animals instead of the usual six individuals and although temperature registration inside the transportation boxes indicated maximum temperatures of 25.5 °C, which is only slightly higher than the optimum comfort zone temperature for rats of 21–23 °C [17]. Usually, the animals are bred and housed at a constant temperature of 22 ± 1 °C during their lives and are not exposed to any (either cold or heat) temperature stress. Further, since the animals drink (and eat) very little during transfer, they have very limited means of regulating their body temperature while the different stressors the animals experience during the transfer are likely to accumulate and to induce a rise in body temperature of rats [64]. Due to methodological considerations we did not include measurements of core temperature in the animal itself in the current study, but restricted our assessment to cage temperature. However, since body temperature is considered highly relevant for potential transfer effects, this parameter was further investigated in a follow up study [65] A sex effect was found in heart rate as well (Figure 3a, b), with females having an overall higher heart rate than males. Quite unexpectedly though, in males and females the transfer effects on heart rate seemed to be opposing: transferred females showed higher heart rate levels after transfer than non-transferred control females, while in males the transferred animals showed lower heart rate levels than the non-transferred control males, similar to the previous study. Although Azar *et al*. found sex and treatment × sex differences in heart rate, and also in heart rate response to a stressor in rats, they did not report any opposing sex-treatment interactions [66]. In this study, however, the effects of transfer on heart rate seem to indicate that female rats may have a different physiological response to transfer than males. The lapse of heart rate after transfer indicates allostatic stabilization in approximately 1 week in males and in just over 2 weeks in females, although no return to before-transfer levels is observed, which makes it somewhat difficult to point out the exact moment of recovery based on heart rate solely. Considered together with activity, this information would strengthen the advice to acclimatize rats for a period of two weeks.

## 5. Conclusion

Extending the effects found in male rats in a previous study, significant transfer effects were found in both male and female rats. It surely needs to be mentioned that the study was performed at an age of the animals at which they are still physically developing, which makes the observed lack of stable baselines as observed in almost all parameters somewhat difficult to interpret. However, transferring experimental animals at about this age is daily practice and, in consequence, is the difficulty of determining a definite acclimatization period. Nevertheless, some clear transfer effects were found, such as an increase in heart rate and activity in female rats that may be used to frame a useful acclimatization period.

This study shows that acclimatization processes can be sex-specific and researchers surely should take this specificity into consideration when planning acclimatization periods after transport of their experimental animals. Based on the current study, and taking the results of the previous study into consideration, we would suggest that two weeks are likely to be sufficient for stabilization of the studied parameters after transfer, while the often recommended one week clearly would be insufficient. We would advise to postpone experimental procedures in males until at least 8 days and in females at least two weeks after arrival of the animals from a supplier and, further, timely (at least 2 days before starting) move animals to experimental units if this requires internal transfer.

## Abbreviations

ANOVA: analysis of variance
auc: area under the curve
BS: blood sample
CORT: plasma corticosterone
DAT: day after transfer
DBT: day before transfer
